# Delayed epidural transplantation of human induced pluripotent stem cell-derived neural progenitors enhances functional recovery after stroke

**DOI:** 10.1038/s41598-017-02137-w

**Published:** 2017-05-16

**Authors:** I-Hui Lee, Shiang-Suo Huang, Ching-Yu Chuang, Ko-Hsun Liao, Li-Hsin Chang, Chia-Chi Chuang, Yu-Shih Su, Hung-Jui Lin, Jui-Yu Hsieh, Shu-Han Su, Oscar Kuang-Sheng Lee, Hung-Chih Kuo

**Affiliations:** 1 Department of Neurology, Neurological Institute, Taipei Veterans General Hospital, Taipei, Taiwan; 2 Institute of Brain Science, National Yang-Ming University, Taipei, Taiwan; 3 Department of Pharmacology and Institute of Medicine, Chung-Shan Medical University, Taichung, Taiwan; 4 Department of Pharmacy, Chung Shan Medical University Hospital, Taichung, Taiwan; 5 Stem Cell Program, Institute of Cellular and Organismic Biology, Academia Sinica, Taipei, Taiwan; 6 Genomics Research Center, Academia Sinica, Taipei, Taiwan; 7 Institute of Microbiology and Immunology, National Yang-Ming University, Taipei, Taiwan; 8 Institute of Clinical Medicine, National Yang-Ming University, Taipei, Taiwan; 9 Department of Orthopaedic Surgery, Taipei City Hospital, Taipei, Taiwan; 10 Stem Cell Research Center, National Yang-Ming University, Taipei, Taiwan

## Abstract

Induced pluripotent stem cell-derived neural progenitor cells (iPSC-NPCs) are a promising source of tailor-made cell therapy for neurological diseases. However, major obstacles to clinical use still exist. To circumvent complications related to intracerebral administration, we implanted human iPSC-NPCs epidurally over the peri-infarct cortex 7 days after permanent middle cerebral artery occlusion in adult rats. Compared to controls, cell-treated rats showed significant improvements in paretic forelimb usage and grip strength from 10 days post-transplantation (dpt) onwards, as well as reductions in lesion volumes, inflammatory infiltration and astrogliosis at 21 dpt. Few iPSC-NPCs migrated into rat peri-infarct cortices and exhibited poor survival in tissue. To examine the paracrine therapeutic mechanisms of epidural iPSC-NPC grafts, we used transmembrane co-cultures of human iPSC-NPCs with rat cortical cells subjected to oxygen-glucose deprivation. Compared to other human stem cells, iPSC-NPCs were superior at promoting neuronal survival and outgrowth, and mitigating astrogliosis. Using comparative whole-genome microarrays and cytokine neutralization, we identified a neurorestorative secretome from iPSC-NPCs, and neutralizing enriched cytokines abolished neuroprotective effects in co-cultures. This proof-of-concept study demonstrates a relatively safe, yet effective epidural route for delivering human iPSC-NPCs, which acts predominately through discrete paracrine effects to promote functional recovery after stroke.

## Introduction

Induced pluripotent stem cells (iPSCs) are produced from somatic cells by overexpression of Sox2, Oct4, c-Myc, and Klf4^1–4^, and exhibit characteristics of embryonic stem cells (ESCs), including self-renewal and ability to differentiate into cells of all three embryonic germ layers^5^. These cells provide an important advance for patient-specific disease investigations and an unprecedented cell source for regenerative medicine^6–8^. However, the risks of *in vivo* tumorigenesis^9–11^ and immunogenicity^12, 13^ are major obstacles to clinical application of iPSC-based therapy, in contrast to approaches using tissue-specific stem cells^14^. Importantly, delivery route and iPSC differentiation state before engraftment are major determinants of therapeutic efficacy^15^. Intracerebral transplants of iPSCs have been found to sometimes form teratomas, which occur even more frequently in post-ischemic brains^16, 17^. These adverse effects may be reduced by utilizing extraparenchymal delivery routes^18^. Additionally, grafting late differentiation stage, iPSC-derived neural progenitor cells (iPSC-NPCs), following spinal cord injury, was found to promote functional recovery without teratoma formation^19, 20^. Intrastriatal engraftment of iPSC-NPCs in adult rats, after stroke, has been shown to enhance functional recovery without teratoma formation for at least 4 months^21^. In this study, the grafted cells were thought to act through complex, predominantly paracrine effects, rather than neuronal replacement. However, the secreted therapeutic components of transplanted iPSC-NPCs that promote stroke recovery have not been described.

Neural stem cells (NSCs) have been considered optimal, but clinically inaccessible for use in restorative treatments of stroke^22^. A randomized, controlled phase II trial in chronic stroke patients comparing stereotactic intracerebral implantation of a human NSC line (NT2/D1, Layton BioScience, Inc., CA, USA) with rehabilitation alone showed insignificant differences between treatments in the European Stroke Scale motor scores after 6 months^23^. Another phase II trial using a human NSC line (CTX0E03, ReNeuron Ltd., UK) is ongoing^24^. NSC-based therapy is likely to involve multiple mechanisms, including trophic support, neuroprotection, immunomodulation, angiogenesis and axonal sprouting/regeneration, although the molecular mechanisms underlying these effects remain unclear^25^. Intravenous infusion of NSCs 3 days after transient middle cerebral artery occlusion (MCAO) in mice has been shown to confer post-ischemic neuroprotection involving anti-inflammatory and anti-astroglial mechanisms^26^. Nevertheless, cells injected intravenously are almost always trapped in the lungs and are rarely found in the brain^27^. Aside from intravenous infusion, intracerebral implantation of a human NSC line (CTX0E03), performed better than intraventricular delivery, with regard to graft survival and functional recovery after MCAO in adult rats^28^. Additionally, a biopolymer hydrogel matrix was shown to provide a desirable vehicle for intraparenchymal or extraparenchymal administration of cells in an otherwise inhospitable stroke brain^29^. Here, we investigated the strategy of epidural transplantation of human iPSC-NPCs, via biopolymer fibrin glue, in an adult stroke rat model. The paracrine therapeutic mechanisms of the iPSC-NPC transplants were further investigated using a transmembrane co-culture system with cortical cells subjected to oxygen-glucose-deprivation (OGD). This culture system was used to compare protective effects of multiple human stem cell types and identify secreted factors from iPSC-NPCs that confer neuroprotection.

## Results

### Efficient generation of neural progenitors from human iPSCs

**Figure 1:**
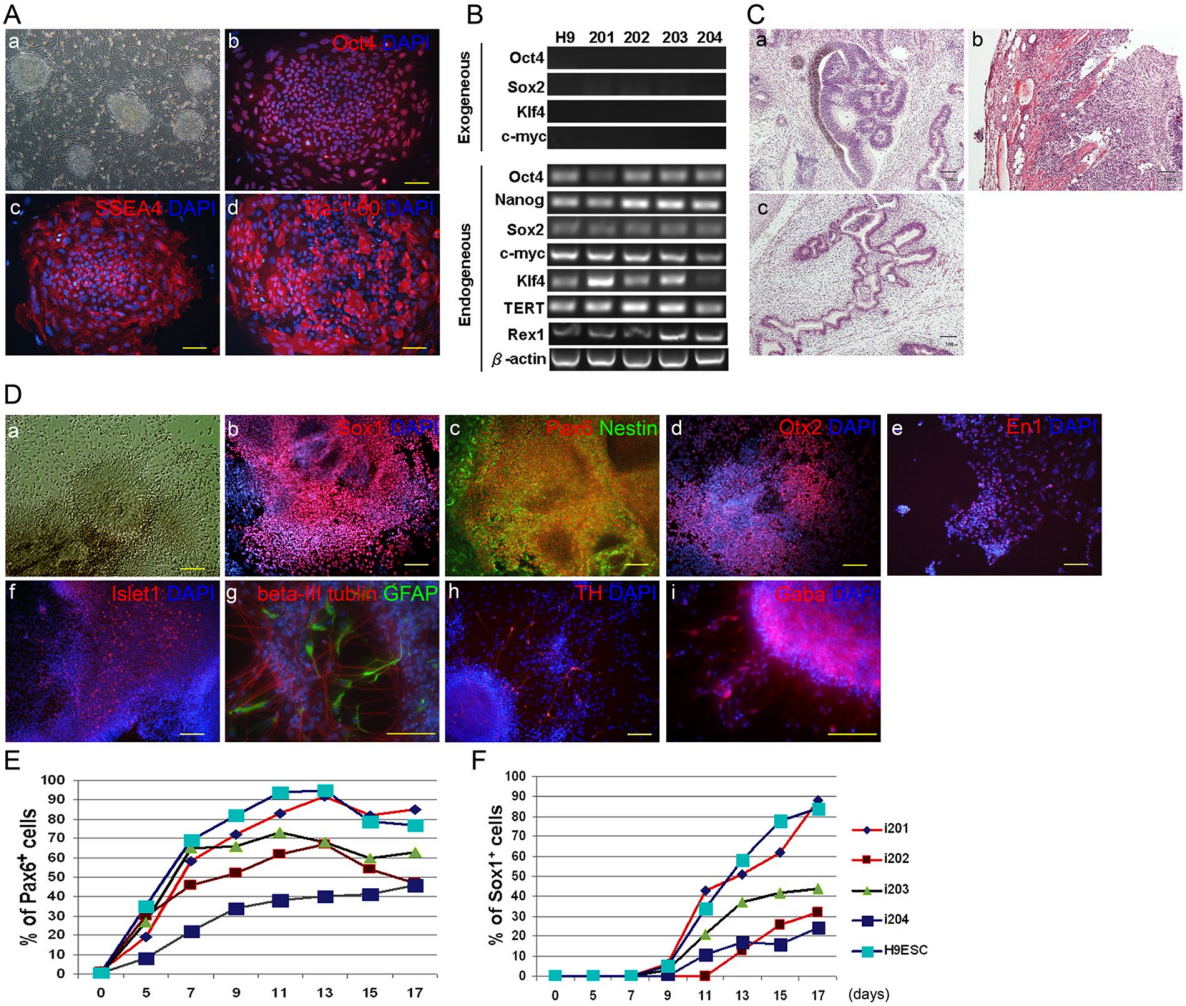
Derivation, characterization, and neural differentiation of human iPSCs. (**A**) Phase-contrast (a) and immunocytochemistry of colonies derived from human iPSCs (clone 201), which express ESC markers, including Oct4 (b), SSEA4 (c), and TRA-1-60 (d). Nuclei were counterstained with DAPI. (**B**) RT-PCR analysis of the expression of retroviral transgenic (exogenous) and endogenous Oct4, Sox2, Klf4, and c-Myc in 4 putative iPSC clones (clones 201–204) and the human ESC line H9. Note that the silencing of all 4 transgenes and the reactivation of their endogenous counterparts was observed in all of the iPSC clones. (**C**) H&E staining of teratomas from iPSCs (clone 201) in NOD-SCID mice containing ectodermal (a), mesodermal (b), and endodermal (c) lineages. (**D**) Morphology and immunoreactivity of iPSCs (clone 201) and the derivative iPSC-NPCs in a time sequence: phase contrast image of neural progenitors in a rosette-like structure (a); expression of early neural progenitor markers Sox1 (b) and Pax6 (c); region-specific forebrain marker Otx2 (d), midbrain marker En1 (e), and hindbrain marker Islet1 (f); after one month of differentiation, partial expression of the neuronal marker ß-III tubulin and the astrocytic marker GFAP (g), neuronal TH (h), and neuronal GABA (i). (**E**,**F**) FACS analysis of the proportion of Pax6- and Sox1-positive neural progenitors at various stages of differentiation (x-axis: days *in vitro*) from different iPSC clones (i201–204) and H9 ESCs. The data are averaged from 3 replicates for each clone. Scale bar (**A**,**C**,**D**) = 100 µm.

We transduced human foreskin fibroblasts with retroviral vectors encoding Oct4, Sox2, Klf4 and c-Myc. Self-renewing cell colonies resembling ESC colonies (Fig. 1Aa) began to emerge along with partially reprogrammed granular cell colonies in suspension culture 12–15 days after viral transduction. After further expansion, 4 putative iPSC clones were selected on the basis of their morphological traits and characteristic growth patterns^30, 31^. The putative iPSCs were found to express the ESC markers SSEA4 and TRA-1-60 as well as the pluripotency marker Oct4 by immunocytochemistry (Fig. 1A). Furthermore, qPCR analysis revealed that the expression of all exogenous reprogramming factors (Oct4, Sox2, Klf4, and c-Myc) was silenced, whereas the expression of their endogenous counterparts was reactivated in all of the tested putative iPSC clones. In addition, similar observations were made for the other pluripotency genes (Fig. 1B). After injection of the putative iPSC clones into the NOD-SCID mice, we identified teratomas that contained cell lineages from all 3 embryonic germ layers (Fig. 1C), indicating that these cell clones were fully reprogrammed iPSCs.

To induce neural differentiation, the human iPSC clones were subjected to a two-step differentiation procedure that we had previously employed for human ESCs^32^. First, clumps of iPSC colonies were cultured in suspension for embryoid body (EB) formation. Similar to results with ESCs, the iPSCs formed spherical cell aggregates after 2–3 days in these conditions. Second, after being re-plated onto a culture surface under serum-free conditions, columnar cells, resembling early neural epithelial cells, began to emerge after 6–10 days. These cells formed neural tube-like rosette structures around day 8–12 (Fig. 1Da). Furthermore, the iPSC-derived neural rosettes homogeneously expressed the early neural progenitor markers SOX1, PAX6, and Nestin, and then later, they expressed the regional neural progenitor markers Otx2, En1, and Islet1 (Fig. 1Db–f). After one month of exposure to differentiation medium without bFGF, the iPSC-derived neural progenitors expressed neuron-specific ß-III tubulin, some tyrosine hydroxylase, and gamma-aminobutyric acid (GABA) (Fig. 1Dg–i). Taken together, these results indicate that the neural differentiation potential of human iPSCs resembles that of their ESC counterparts. Although all 4 human iPSC clones gave rise to neural epithelial cells, we noted that the efficiency of developing neural phenotypes varied among the clones. We temporally quantified the proportion of iPSC- and ESC-derived PAX6- or SOX1-expressing neural progenitors by flow cytometric analysis. The percentage of PAX6-expressing cells from the iPSC clones gradually increased from day 5 onwards and reached peak levels from day 11–13 (Fig. 1E). Unlike the PAX6-expressing population, the SOX1-expressing population was not evident until day 9, and SOX1 expression reached a plateau at day 17 (Fig. 1F). Importantly, the proportions of the PAX6- or SOX1-expressing populations varied among the different iPSC clones, and clone i201 was the only clone that was able to generate neural precursors *in vitro* with a similar efficiency to that of ESCs.

### Delayed epidural engraftment of iPSC-NPCs after MCAO promoted functional recovery and ameliorated cerebral damage through paracrine effects

**Figure 2:**
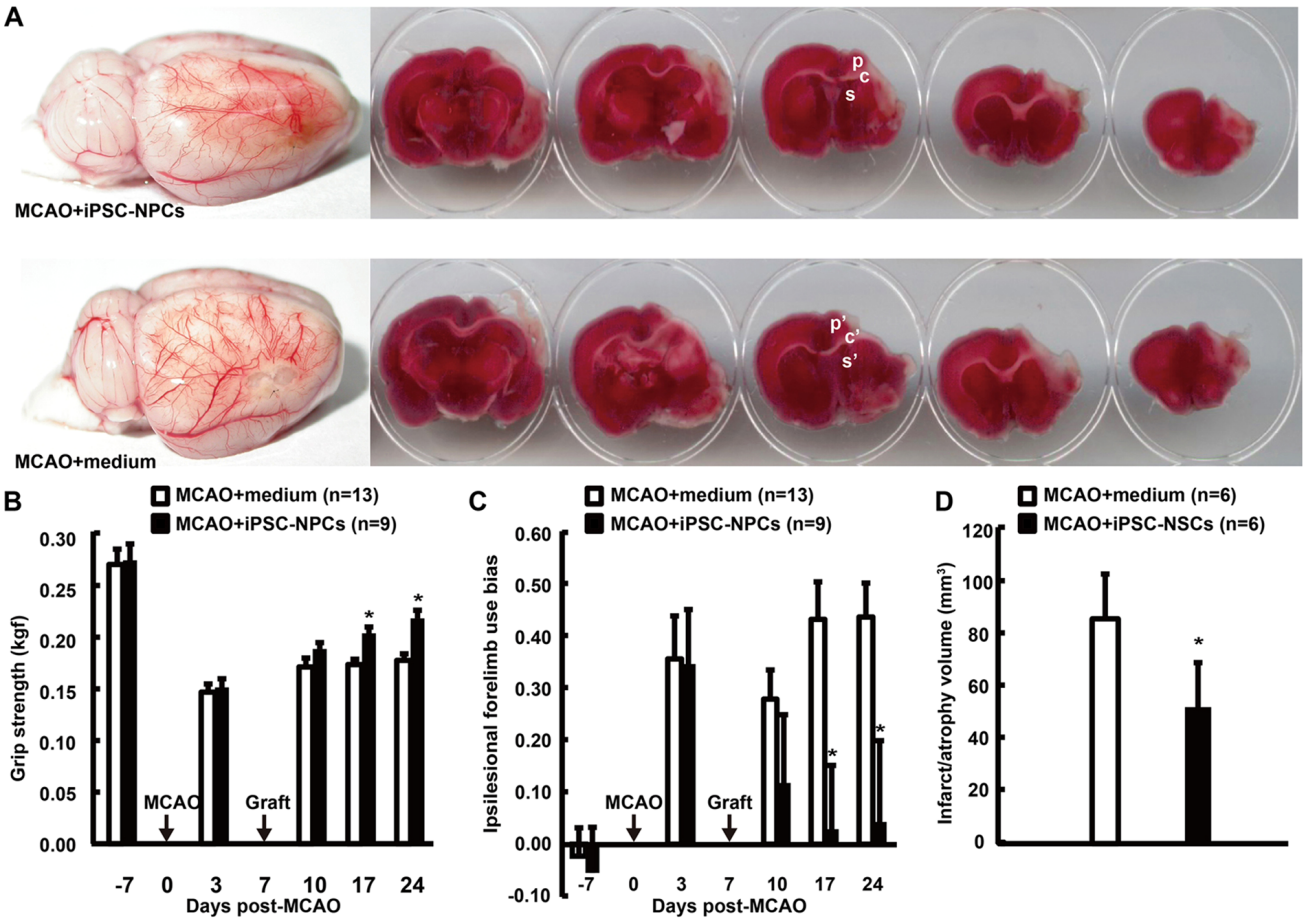
iPSC-NPC engraftment reduced the lesion volume and improved functional recovery after MCAO. (**A**) Representative brains and derivative slices with vital staining from stroke rats with one-week delayed transplantation of iPSC-NPCs or vehicle via local epidural fibrin glue. At 21 days post-transplantation (dpt), note that the white cortical/subcortical lesions and cavities decreased with iPSC-NPC engraftment. The behavioral performance of left paretic forelimbs were progressively monitored using the grip strength test (**B**) and the cylinder test (**C**). The iPSC-NPC group showed functional improvement from 10 dpt compared with the control group. (**D**) The histological analyses at 21 dpt showed that the iPSC-NPC group had reduced lesion volume compared with the controls. p, p’: peri-infarct cortex; c, c’: corpus callosum; s, s’: subventricular zone; indicating the locations of representative pictures in Fig. 3. The results are expressed as mean ± standard error of the mean (SEM). *P < 0.05.

Before cell transplantation at 3 days post-MCAO, the baseline behavioral measures of the paretic forelimb were similar between the two groups (Fig. 2B,C). After transplantation at 7 d post-MCAO, the cell-transplanted rats exhibited a progressive improvement in grip strength and the frequency with which they used their left paretic forelimb. Performance became significantly better than the control rats beginning at 10 days post-transplantation (dpt) and this difference persisted throughout the study (Fig. 2B,C, *P* < 0.05 at 17 d and 24 d post-MCAO). The grip strength of the right unaffected forelimb did not differ between the two groups (data not shown). At 21 dpt, histological analysis showed that permanent MCAO resulted in severe cortical and subcortical lesions (Fig. 2A). On the basis of hematoxylin and eosin staining, the cell-transplanted rats demonstrated a significantly reduced lesion volume compared with the controls (Fig. 2D, *P* < 0.05), which was likely related to their functional improvement.

**Figure 3:**
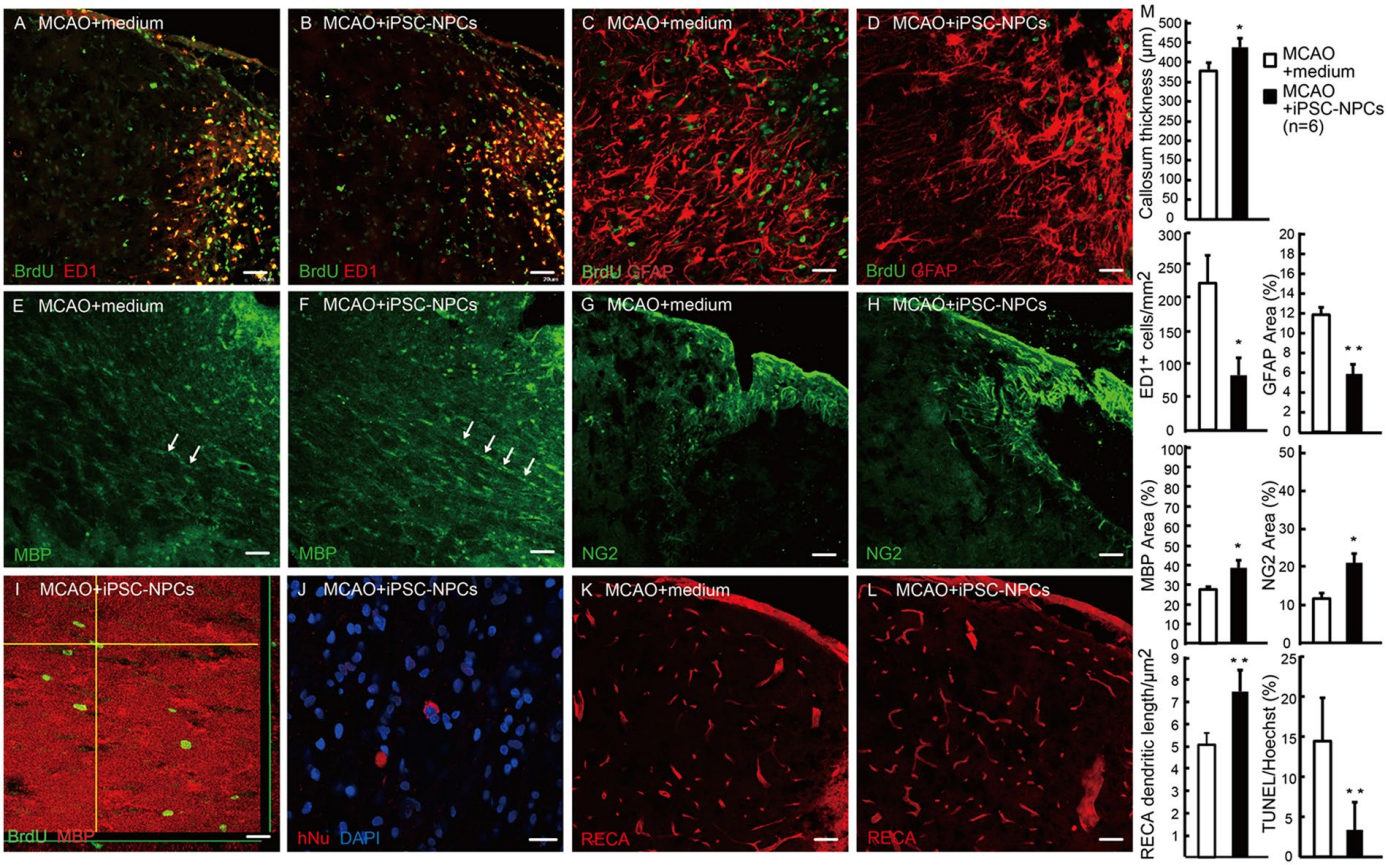
Epidural iPSC-NPCs grafts ameliorated inflammation and astrogliosis, and augmented endogenous oligodendrocyte progenitor cells, white matter tract integrity, and angiogenesis. Immunohistochemistry at 21 days post-transplantation. (**A**,**B**) In the peri-infarct cortex, labeled as p, p’ in Fig. 2A, BrdU-labeled cells were mainly identified as ED1-immunoreactive microglia, macrophages or hematogenous monocytes, and to a lesser extent, astrocytes (**C**,**D**) and oligodendrocytes (**I**). The inflammatory infiltration was significantly ameliorated in the iPSC-NPC group (**M**), as was the GFAP-immunoreactive astrogliosis (**C**,**D**,**M**). (**E**,**F**) Moreover, in the iPSC-NPC group, myelination (MBP-immunoreactive oligodendrocytes) in the paramedium corpus callosum, labeled as c, c’ in Fig. 2A, was better preserved (**M**), and some endogenous nascent oligodendrocytes were observed by BrdU-pulse labeling (**I**). NG2-positive oligodendrocyte progenitors were robustly increased in the penumbra in the iPSC-NPC group (**G**,**H**,**M**). Few exogenous human nuclei-positive cells migrated and survived in the peri-infarct cortex following an epidural transplantation (**J**). There was also increased RECA-positive endothelial vasculature in the peri-infarct cortex of the cell-transplanted brain (**K**,**L**,**M**). (**M**) Between-group comparisons of immunohistochemical quantifications. Scale bar (**A**,**B**,**E–H**,**K**,**L**) = 50 µm; (**C**,**D**) = 25 µm; (**I**,**J**) = 10 µm. *P < 0.05; **P < 0.01.

The epidural engrafted iPSC-NPCs, marked by the colocalization of human nuclear antigen and DAPI, had poor survival in the peri-infarct cortex (Fig. 3J, mean ± standard deviation: 1.83 ± 1.04 cells per section per iPSC-NPC grafted rat, absent in controls), which suggests that the observed functional recovery was likely due to paracrine effects. Importantly, no tumor formation was noted in any cell-transplanted rat. The ischemic boundary zone showed accumulation of BrdU-positive cells that were mostly identified as ED1-immunoreactive resident microglia or blood-derived monocytes (Fig. 3A,B), which would be expected to rapidly respond to ischemic damage and phagocytize cellular debris. To a minor extent, the BrdU-labeled cells included GFAP-immunoreactive astrocytes (Fig. 3C,D) or MBP-immunoreactive oligodendrocytes within white matter tracts (Fig. 3I). Very few peri-infarct NG2-immunoreactive oligodendrocyte progenitor cells (OPCs), peri-infarct RECA-immunoreactive endothelial cells, and doublecortin (DCX)-immunoreactive neural progenitors from the subventricular zone were also noted (data not shown). The epidural iPSC-NPC transplantation attenuated ED1 infiltration, astrogliosis, and apoptosis (Fig. 3A–D,M). Meanwhile, transplanted animals showed enhanced angiogenesis, thicker corpus callosum, and increased number of MBP-positive (re)myelinating oligodendrocytes (Fig. 3E,F,I,M) and NG2-expressing OPCs (Fig. 3G,H,K,L,M), which were not derived from migratory neuroblasts from the subventricular zone. Parenchymal OPCs express a complex set of voltage-gated channels, receive glutamatergic and/or GABA-ergic synaptic input from neurons, provide trophic support, and display distinct progenitor activities in the adult brain particularly after injury^33, 34^. These latent progenitor cells may differentiate into myelinating oligodendrocytes or even a few neurons^35^ if properly induced. However, their functional role after stroke remains largely unknown. Distal to the epidural transplants, numbers of DCX-positive neural progenitors in the subventricular and subgranular zones were not different between the groups (data not shown).

### Secreted factors from iPSC-NPCs for OGD-injured cortical cells

**Figure 4:**
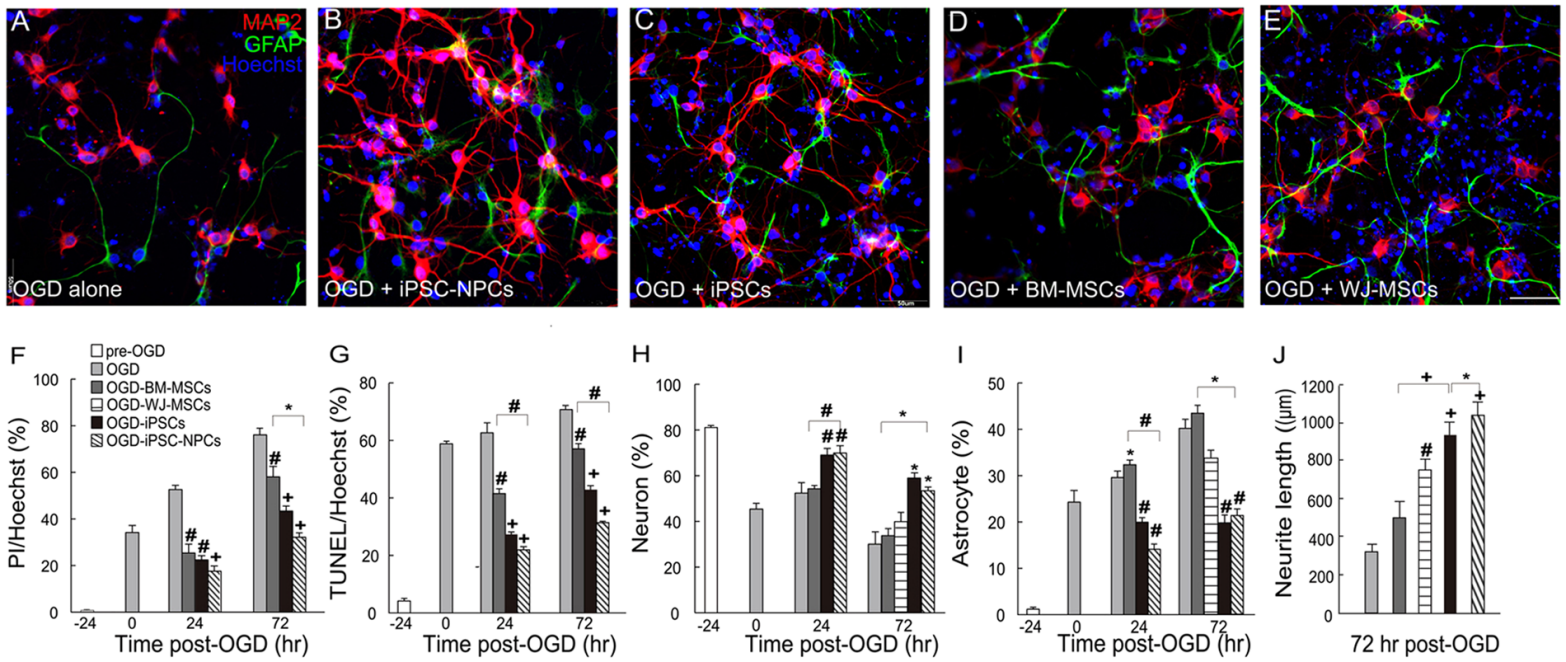
Preferential neuroprotection after oxygen-glucose deprivation (OGD) injury by co-culture with iPSC-NPCs. (**A**) Immunocytochemistry of the rat primary cortical cells subjected to OGD alone and those with transmembrane co-cultures of human (**B**) iPSC-NPCs, (**C**) iPSCs, (**D**) MSCs from bone marrow (BM-MSCs), and (**E**) umbilical cord Wharton’s jelly (WJ-MSCs) at 72 hours post-OGD. The neuronal marker MAP2 is red; the astroglial marker GFAP is green; Hoechst nuclear staining is blue. (**F**) Quantification of the percentage of cell death by propidium iodide (PI) staining, (**G**) apoptotic cells by TUNEL assays, (**H**) the percentage of surviving neurons, (**I**) the percentage of surviving astrocytes, and (**J**) total neurite length at different time points post-OGD. Note that the co-cultures with iPSC-NPCs or iPSCs showed markedly reduced cell death and apoptosis and reduced astrogliosis compared to co-cultures with MSCs from different tissue origins. The results are expressed as mean ± SEM. Scale bar (**A**–**E**) = 50 µm. *P < 0.05; ^#^ *P* < 0.01; ^+^ *P* < 0.001.

To elucidate paracrine therapeutic mechanisms of epidural iPSC-NPC grafts, we simulated the spatial arrangement of cortex and graft with a membrane delimited co-culture system, consisting of iPSC-NPCs on one side of the membrane and OGD-injured cortical cells on the other side. Given that the human iPSC-NPCs or other stem cell types were cultured separately in the upper chambers of the OGD co-cultures, their beneficial effects, if any, should arise from secretion of paracrine factors rather than from direct contact. The primary cortical cultures were severely injured after 24 hours of OGD treatment, and cell death and apoptosis increased with time to more than 70% at 72 hours post-OGD (Fig. 4A). However, after 72 hours of co-culture with the iPSC-NPCs, iPSCs, umbilical cord Wharton’s jelly-derived mesenchymal stem cells (WJ-MSCs) or MSCs from bone marrow (BM-MSCs) following OGD, significant reductions in cell death and apoptosis were observed compared with the OGD-injured alone group. The effects were particularly pronounced in cells co-cultured with iPSC-NPCs and iPSCs (Fig. 4A–G). The surviving neuronal population was robustly increased in the iPSC-NPC and iPSC groups, but not in the BM-MSC or WJ-MSC groups (Fig. 4H). Furthermore, astrogliosis was markedly attenuated in the iPSC-NPC and iPSC groups. However, astrogliosis in the BM-MSC or WJ-MSC groups were similar to the OGD-injured-alone group (Fig. 4I). The total neurite length at 72 hours post-OGD was most significantly increased by co-culture with iPSC-NPCs, followed by iPSCs and WJ-MSCs, compared with the OGD-injured alone group. The BM-MSC co-cultures did not show obvious increases in the total neurite outgrowth compared with the OGD control (Fig. 4J).

**Figure 5:**
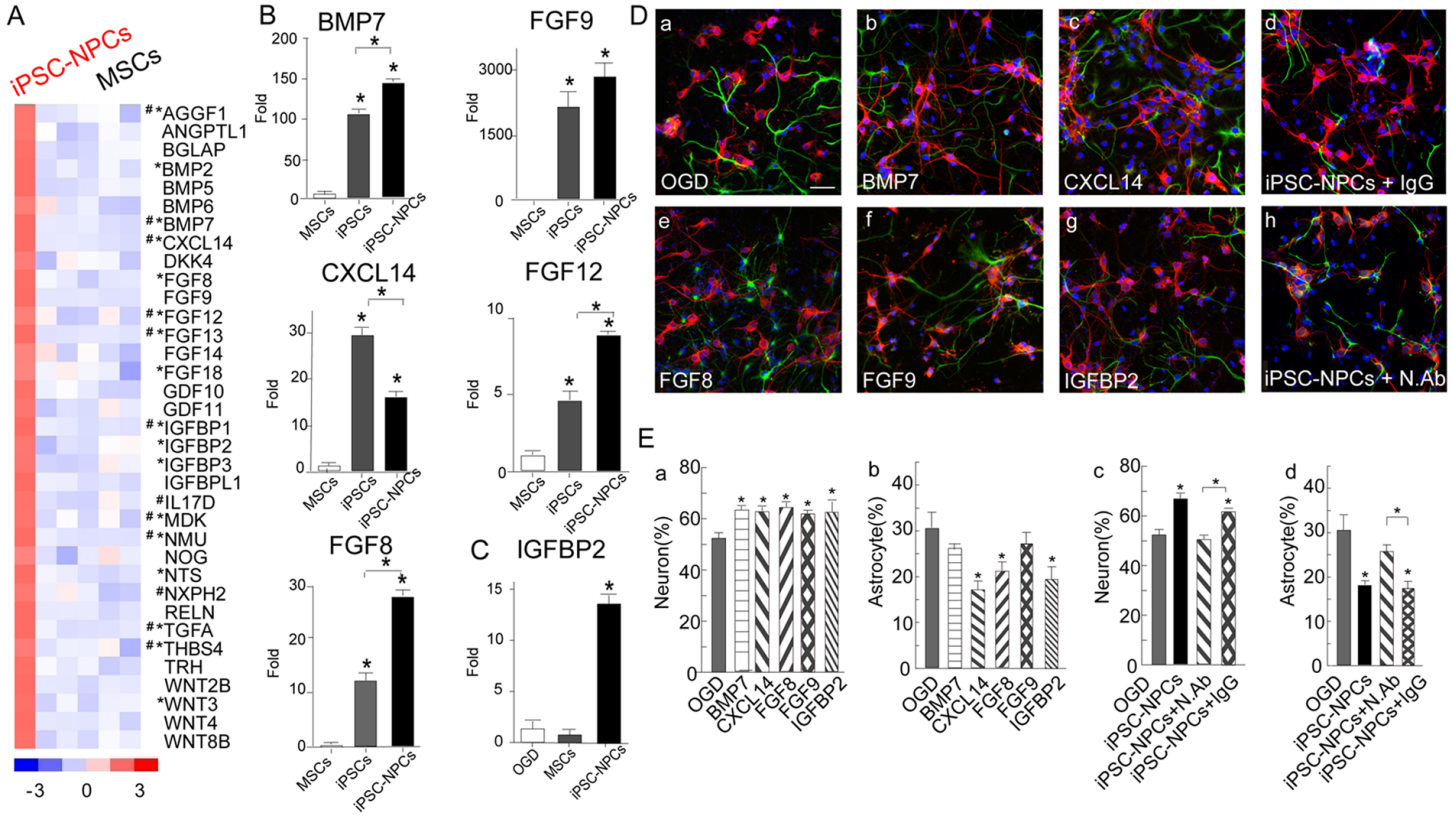
The unique secretome from human iPSC-NPCs. (**A**) A heat map shows differences between the secretomes of iPSC-NPCs (Lane 1, clone 201, GEO accession GSE82101, http://www.ncbi.nlm.nih.gov/geo/query/acc.cgi?acc=GSE82101) and BM-MSCs (Lane 2–6). Genes with # are overexpressed in the iPSCs; genes with * have been previously shown to be overexpressed in other iPSCs compared with BM-MSCs according to published array data. (**B**) Validation of the microarray data by RT-qPCR confirmed the upregulated gene expression of BMP7, CXCL14, FGF12, FGF9, and FGF8 in iPSCs and iPSC-NPCs compared with BM-MSCs. The results are expressed as mean ± standard deviation (SD). (**C**) Cytokine array analysis shows that the supernatants from iPSC-NPC co-cultures were exclusively enriched with IGFBP2 compared to those from BM-MSCs or OGD-injured cultures (Supplementary Fig. 1). (**D**) Immunocytochemistry of OGD-injured cortical cells alone (a) and those treated with BMP7 (100 ng/ml, b), CXCL14 (10 ng/ml, c), FGF8 (100 ng/ml, e), FGF9 (10 ng/ml, f), and IGFBP2 (100 ng/ml, g) shows neuroprotection of the individual cytokines. Note that a neutralizing antibody cocktail (N. Abs, h), including anti-BMP7, anti-CXCL14, anti-FGF9, anti-FGF8, and anti-IGFBP2, significantly abolished the neuroprotection effects from iPSC-NPCs compared with the control antibodies (IgG, d, also Ec,d). (**E**) The results are expressed as mean ± SEM. Scale bar (**D**) = 50 µm. *P < 0.05.

To identify the soluble factors responsible for these neuroprotective effects, we compared the gene expression profiles of the human iPSC-NPCs, iPSCs and BM-MSCs according to an established statistical pipeline^36, 37^. A comparative transcriptome analysis revealed that the transcripts of 35 secreted factors were abundantly overexpressed in the iPSC-NPCs, but not in the BM-MSCs (Fig. 5A). Of these 35 secreted factors, 12 were also commonly overexpressed in the iPSCs (#; positive false discovery rate threshold *q* < 0.01 and fold change above 1.5-fold). In addition, 17 of the 35 secreted factors were shown to be overexpressed in other published studies of iPSCs compared with MSCs (*q < 0.001 and fold change above 2-fold). The differential expression levels of the selected factors (BMP7, CXCL14, FGF8, FGF9, FGF12) among the iPSCs, iPSC-NPCs, and BM-MSCs were verified by RT-qPCR (Fig. 5B; primer sequences listed in Supplementary Table 2) and western blotting analyses (Supplementary Fig. 2). Furthermore, using human cytokine arrays, we compared the cytokine profiles in the supernatants from the OGD-alone group and the co-cultures with iPSC-NPCs or BM-MSCs. We found that the expression level of IGFBP2 was exclusively high in the iPSC-NPC co-culture supernatants (Fig. 5C), whereas MCP1 expression was significantly elevated in both the iPSC-NPC and BM-MSC co-culture supernatants (Supplementary Fig. 1). To identify the cellular origins of the cytokines, we observed that the mRNA level of IGFBP2 was upregulated in the iPSC-NPCs, but not in the BM-MSCs (Fig. 5A). MCP1 mRNA was highly expressed in the BM-MSCs, but not in the iPSC-NPCs (Supplementary Fig. 1), suggesting that IGFBP2 was primarily secreted by the co-cultured human iPSC-NPCs and that MCP1 was secreted by the human BM-MSCs and rat OGD cells co-cultured with iPSC-NPCs. To confirm the beneficial effects of the secreted factors from human iPSC-NPCs, we tested 5 commercially available cytokines (BMP7, CXCL14, FGF8, FGF9, and IGFBP2) that were upregulated in the iPSC-NPCs. After treating the OGD-subjected cortical cells with the individual cytokines for 72 hours, we found that each cytokine conferred neuroprotection compared with the OGD-injured alone group (Fig. 5D,Ea). Notably, among the cytokines, CXCL14, FGF8, and IGFBP2 markedly reduced astrocytosis (Fig. 5Eb). A cocktail of the 5 cytokines conferred similar neuroprotective effects as the iPSC-NPCs, however, no obvious synergistic effects were found (data not shown). Conversely, a cocktail of neutralizing antibodies against the 5 cytokines abolished the neuroprotection and astroglial inhibitory effects in the iPSC-NPCs co-cultures compared with OGD-injured alone, or iPSC-NPC co-cultures treated with control antibodies (Fig. 5Dd,h, Ec,d).

### The molecular impacts of iPSC-NPCs on OGD-injured cortical cells

**Figure 6:**
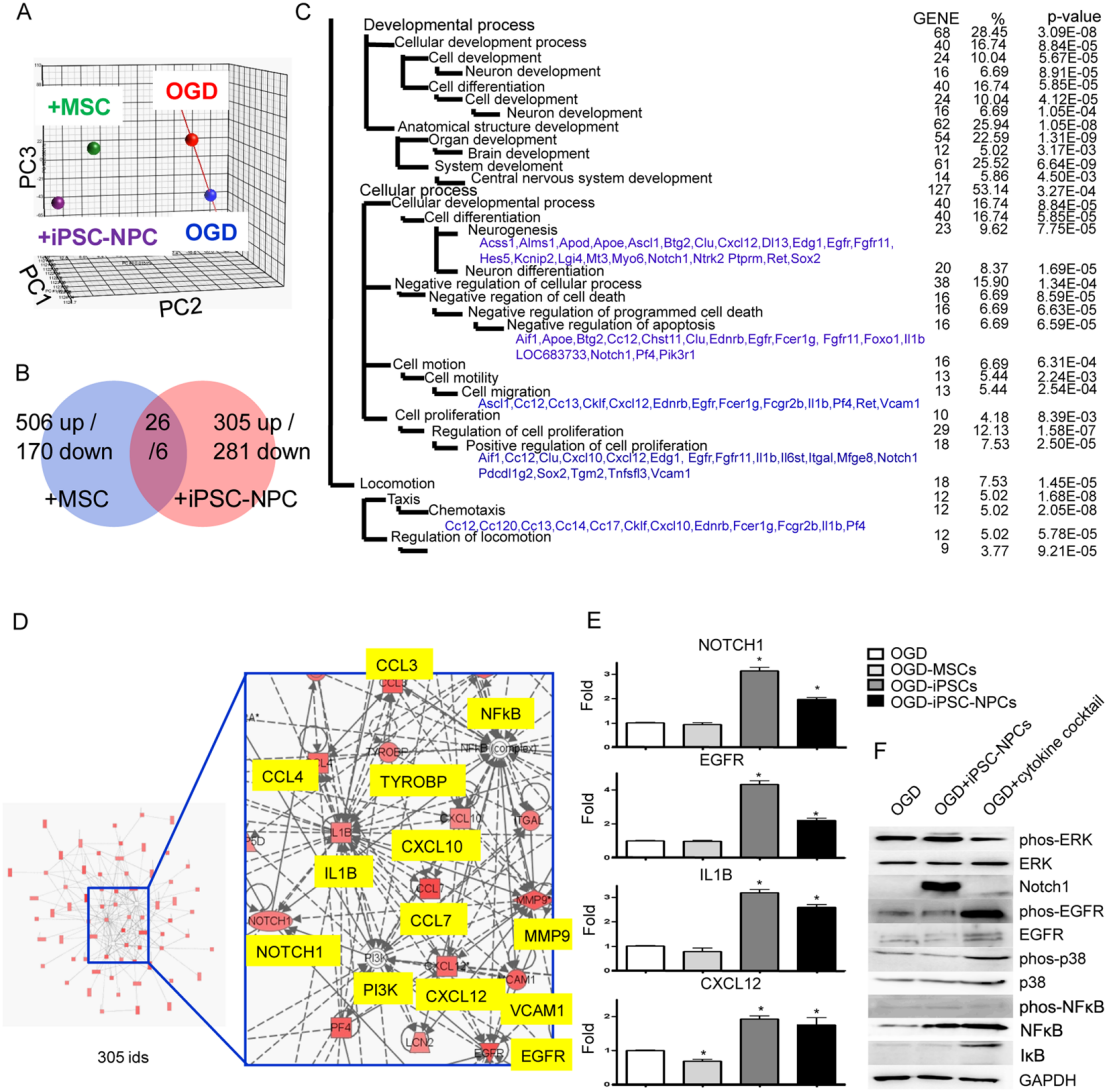
Comparative and interaction network analyses as a framework for iPSC-NPC-induced neuroprotection. (**A**) A principle component analysis plot using the entire set of 54,600 probes represented on the chips. (**B**) A Venn diagram illustrates that 305 probe sets were more abundantly expressed in rat cells co-cultured with iPSC-NPCs. (**C**) Altered biological modules in rat cells co-cultured with iPSC-NPC according to the Gene Ontology database. The significantly enriched number of genes, gene symbols, and *P*-values are listed (*P* < 0.05). (**D**) A functional genetic network composed of the above 305 probe sets. This network is graphically displayed as nodes (gene products) and edges (biological relationships between nodes) mapped by the Ingenuity^TM^ Pathway Analysis tool. The intensity of the node color indicates the degree of upregulation. Left panel: the core region of this gene network, highlighting several hub genes. (**E**) Validation of the array data by RT-qPCR. Mean expression levels of target genes were compared with GAPDH as a control. Each bar represents a different group. The results are expressed as mean ± SD. (**F**) The protein immunoblot analysis of rat cells subjected to OGD-alone and those that were treated with either iPSC-NPCs or the cytokine cocktail.

To investigate the OGD-injured cortical cell response to iPSC-NPC-secreted cytokines, we analyzed the entire rat transcriptome in OGD-injured cortical cells with or without co-cultured iPSC-NPCs or BM-MSCs. The principle component analysis plot illustrates differential gene expression patterns among the rat OGD-injured alone cells and those that were co-cultured with iPSC-NPCs or BM-MSCs (Fig. 6A). Compared with the OGD-injured alone, the OGD-injured rat cells that were co-cultured with iPSC-NPCs had 331 probe sets that were abundantly overexpressed, while those co-cultured with BM-MSCs had 532 probe sets that were overexpressed (with at least 1.5-fold change) (Fig. 6B). Among them, only 26 genes were commonly upregulated in the OGD-injured rat cells that were co-cultured with either iPSC-NPCs or BM-MSCs, indicating that these two stem cell types produce distinct effects in injured cells. The iPSC-NPC co-culture exclusively affected 586 probe sets, including 305 genes that were upregulated and 281 genes that were downregulated (Fig. 6B). We hypothesized that these genes were effectors for the neuroprotective factors secreted by iPSC-NPCs. To gain more insight into the functional consequences of upregulating these genes, the 305 upregulated signature probe sets were searched in the Gene Ontology database to find statistically over-represented functional groups within gene lists. Given that the entire rat transcriptome was represented in the microarray, this analysis was not biased by incomplete coverage due to microarray probe selection. We found that in the presence of iPSC-NPCs, OGD-injured rat cells were significantly enriched with expression of genes involved in neurogenesis and anti-apoptosis (*P* = 7.75 × 10^−5^ and 6.59 × 10^−5^, respectively; Fig. 6C). Among the overexpressed genes, 23 were involved in neurogenesis, including Notch homolog 1 (Notch1), epidermal growth factor receptor (Egfr), and Cxcl12. Enrichment of these gene products suggests that the corresponding ligands of these receptors and stemness genes may contribute to the survival or proliferation of rat neurons co-cultured with iPSC-NPCs. To understand how these enriched gene products interact and affect cellular function, we performed a genetic network analysis for the signature genes using the Ingenuity^TM^ Pathway Analysis. Among the aforementioned 305 probe sets, a core network consisting of 70 genes was identified; these included Notch1, Egfr, interleukin-1 beta, Cxcl12, matrix metallopeptidase 9, and vascular cell adhesion molecule 1. These genes exhibit high interconnectivity and play crucial biological roles as ‘hub’ genes^38^ (Fig. 6D). Phosphatidylinositol 3-kinases (PI3K) and nuclear factor kappa B (NFκB) were not present in the filtered gene lists (indicated with white color in Fig. 6D). The expression levels of hub genes in the rat OGD-injured alone, and those co-cultured with iPSC-NPCs, iPSCs, or BM-MSCs were verified by RT-qPCR (Fig. 6E). In parallel, the protein expression profiles showed that Notch1 was robustly upregulated in the OGD-injured cells co-cultured with iPSC-NPCs, but not in OGD-alone cells, and much less in cells treated with the five-cytokine cocktail. In contrast, the cytokine cocktail treated OGD-injured cells had marked activation (phosphorylation) of Egfr and p38 mitogen-activated protein kinases (p38 MAPKs) (Fig. 6F). These results suggest that Notch1 may be a crucial downstream effector of secreted signals that are not represented in the 5 cytokines we tested from the iPSC-NPCs. On the other hand, Egfr may be an effector that mediates neuroprotection by the five-cytokine cocktail.

## Discussion

In this study, we demonstrate the beneficial effects of epidural transplantation of late-stage iPSC-NPCs via fibrin glue at subacute stroke stage. This treatment induced significant functional improvements and limited infarct/atrophy, in association with attenuated inflammatory infiltration and astrogliosis. Moreover, iPSC-NPC transplantation augmented endogenous NG2-expressing OPCs, white matter tract integrity, and angiogenesis. Incorporation of iPSC-NPCs into the ischemic boundary zone was scarcely detected, suggesting the beneficial effects were primarily derived from paracrine factors. In a comparable transmembrane co-culture system with OGD-injured cortical cells, we found that iPSC-NPCs exhibited superior neuroprotection and astroglial inhibition compared with iPSCs, BM-MSCs and WJ-MSCs through a unique secretome that promoted Notch1 signaling. The neurorestorative substrates from iPSC-NPCs have not been sufficiently characterized and further studies are needed to elucidate molecular mechanisms of them. For example, further investigation will be required to determine whether the cytokines tested in the culture system (BMP7, CXCL14, FGF8, FGF9, and IGFBP2) play any role in the *in vivo* therapeutic function of the grafts. Importantly, the strategy of utilizing local extraparenchymal delivery of late-stage iPSC-NPCs decreases tumorigenic risk and enhances the functional regenerative response after stroke even beyond the acute therapeutic time window.

Recent studies have highlighted the observation that the reprogramming process and subsequent culture of iPSCs *in vitro* can induce genomic instability and epigenetic abnormalities in these cells^9, 10, 39, 40^. Thus, concerns have been raised about their *in vivo* safety after transplantation. The subdural transplantation of iPSCs with fibrin glue markedly reduced tumorigenic risk compared with intracerebral injection, which leads to almost 100% tumorigenesis after acute murine stroke^18^. Here, we emphasize the need to remove contaminating iPSCs by differentiating cells for at least 2 months into late-stage neural progenitors/precursors. These late-stage iPSC-NPCs were then used for epidural transplantation with biopolymer 1 week after stroke. Epidural transplantation may be preferred due to reasons related to safety and graft focalization. The dura mater is the outermost collagenous layer and the most substantial of the meninges, providing mechanical strength to stabilize the brain position. It has its own blood supply and drainage from a collection of meningeal arteries and veins, which penetrate the dura itself. These unique properties of the dura mater provide a favorable implantation platform for epidural grafts, which will prevent damage to brain tissues or triggering of foreign body reactions that may occur from intracerebral or subdural grafting. Although the intact dura is poorly permeable, following MCAO or stroke, cell and substrate permeability is substantially increased. This is especially true for the region that covers the peri-infarct cortex. Because few xenogeneic iPSC-NPCs survived in the ischemic boundary cortex during progressive functional recovery, we suggest that paracrine effects are the dominant mechanisms underlying the observed functional and histological improvements (Figs 2 and 3). Hence, we did not examine graft-host neuronal connections. Although use of differentiated iPSC derivatives is less of a health risk than use of iPSCs, safety of cell transplantation *per se* is still a major drawback to any iPSC-based cell therapy. An alternative approach is to identify novel therapeutic mechanisms underlying the iPSC-NPC effects and develop cell-free treatments based on these findings. Besides, the recent advances in direct conversion of somatic cells into induced neural progenitor/stem cells (iNPCs/iNSCs) *in vitro* using defined transcription factors^41^ or small molecules^42^, or *in vivo* engineering^43^ provide other means of expanding iNPCs/iNSCs for cell therapy.

Stem cells can secrete abundant trophic factors to assist in brain repair^44^. Similar to stem cells, in our OGD model, using transmembrane co-cultures, iPSC-NPCs exhibited neuroprotective capacity through soluble factors (Fig. 4). Using functional genomics and cytokine array analyses, we compared the factors overexpressed by iPSC-NPCs with those from BM-MSCs (Fig. 5). Due to the lack of commercially available recombinant proteins or neutralizing antibodies, we further investigated only a limited number of cytokines (BMP7, CXCL14, FGF8, FGF9, and IGFBP2) from the full set of 35 enriched cytokines that were found to comprise the iPSC-NPC secretome. All of the 5 cytokines that we further studied have been individually shown to be involved in neuroprotection. BMP7 plays an inductive role in the generation of postnatal cerebellar neurons^45^, and it also promotes a neural regenerative response and improves motor recovery in rodent stroke models^46, 47^. FGF8 has been implicated in rodent CNS development and mesencephalic dopaminergic differentiation^48, 49^. FGF9 can prevent the death of dopaminergic neurons against methyl-phenylpyridinium-induced neurotoxicity^50^. CXCL14 is a potent chemoattractant and activator of dendritic cells^51^, and BRAK/CXCL14 has been shown to regulate GABA-ergic synaptic transmission in the stem cell niche of the adult mouse dentate gyrus^52^. Moreover, IGFBP2 has been shown to be expressed during early postnatal neurogenesis in the mouse hippocampus^53^ and is upregulated in the hippocampus after murine stroke^54^. Nevertheless, IGFBP2 can also promote glioma development and progression^55, 56^. We show here that BMP7, FGF8, FGF9, IGFBP2, and CXCL14 can individually promote neuronal survival and growth after OGD injury, and a blockade of all 5 cytokines markedly abolished the neuroprotective effects in the iPSC-NPC co-cultures. Furthermore, we found that the molecular targets and downstream intraneural signaling that underlie the paracrine effects of the iPSC-NPCs include neuroprotection, anti-apoptosis, cell proliferation and migration (Fig. 6). Notch1 signaling was specifically activated, which mediates NPC proliferation, neuronal survival, axonal growth, and synaptic formation throughout development and into adulthood^57–59^. Of the 35 enriched cytokines that we identified from the iPSC-NPCs, BMP2^60^ and GDF11^61^ may crosstalk with Notch1 signaling. Further studies are needed to identify the complex interactions among the numerous cytokines, cognate receptors, and other crucial components of iPSC-NPC-mediated neuroprotection.

In conclusion, our results demonstrate the therapeutic potential of late-stage human iPSC-NPCs via epidural delivery 1 week after rodent stroke. Importantly, we observed sensorimotor improvement in the iPSC-NPC-transplanted rats from 10 dpt that was accompanied by reduced lesion volume and peri-infarct tissue remodeling. Similarly, *in vitro*, human iPSC-NPCs were superior to other human MSCs at enhancing neuroprotection of OGD-injured cortical cells and ameliorating astrogliosis. This action proceeded through a unique secretome that we identified using comparative genome-wide analysis and functional assays. These results provide insights into the development of stem cell-based therapies for cerebrovascular diseases.

## Methods

### Human materials and animals

Human samples were obtained with written informed consent from tissue donors, in accordance with the protocol approved by the Institutional Research Board (IRB) of Taipei-Veterans General Hospital (IRB No. 97-09-11A). The experiments involving recombinant DNA were performed according to the National Institute of Health guidelines. The animal experiments were approved by the Institutional Animal Care and Use Committee of Taipei-Veterans General Hospital (IACUC 99–167). The authors declare that all methods were performed according to the relevant guidelines established by the oversight boards and agencies.

### Derivation and neural differentiation of iPSCs

The iPSCs from a 20-year-old male donor were generated as previously described^31^. For the step-wise neural differentiation^32^, iPSCs were detached from feeder cells with dispase (0.5 mg/ml, Invitrogen) and transferred onto Ultra-Low adhesion plates in differentiation medium consisting of Dulbecco’s modified Eagle’s medium (DMEM) with 10% fetal bovine serum (FBS) for EB formation. After 6 days in the suspension cultures, the EBs were plated onto gelatin-coated culture dishes to allow for EB attachment. The medium was replaced for 8 weeks of neural differentiation with N2 medium consisting of DMEM/F12 medium supplemented with non-essential amino acids, L-glutamine, ITS supplement (Invitrogen), and human basic fibroblast growth factor (bFGF, 10 ng/ml). To characterize the neural progenitors, cell clusters with early neuroepithelial rosette-like structures were dissected, collected under a dissection microscope, and analyzed by RT-PCR and immunocytochemistry. For the co-cultures or transplantation experiments, the neurospheres from clone i201 were washed and resuspended in serum-free DMEM/F12 medium. The aliquots were adjusted to a concentration of 5 × 10^4^ cells/µl.

### Permanent middle cerebral artery occlusion and delayed iPSC-NPC engraftment

Adult male Long-Evans rats (8 weeks old, 250–300 g body weight) were subjected to a permanent right MCAO as previously described^62^. Animals were anesthetized by an intraperitoneal chloral hydrate injection (Sigma, 450 mg/kg body weight in saline). The body temperature was maintained at 37 ± 0.5 °C with a heating pad. The dura was opened with fine forceps, and the right MCA was ligated with 10-0 monofilament nylon ties under a dissecting microscope (OPMI-1, ZEISS, Germany). The bilateral common carotid arteries were exposed and occluded by microaneurysm clips for 1 hour. One week after the MCAO, the animals were randomly assigned into 2 groups: (1) the iPSC-NPC group, which received 1 × 10^6^ cells per recipient via a total volume of 40 µl epidural fibrin glue (Beriplast P, Germany) prepared before use by mixing 100 mg/ml fibrinogen with 200 KIU/ml aprotinin and 8 mM calcium chloride over the infarct cortex^63^, and (2) the control group, which received 40 µl of epidural fibrin glue containing only the culture medium (n = 9, 13 respectively). All of the animals received a daily injection of 5-bromo-2′deoxyuridine (BrdU, 50 mg/kg/d, Sigma) dose for the first 12 days^64^ after MCAO to label endogenous as well as xenogeneic cell proliferation, and daily cyclosporin A (10 mg/kg) after the transplantation until the animals were sacrifice at 14 days or 28 days post-MCAO. The iPSC-NPC xenografts were recognized by endogenous human nuclear antigen and immunohistochemistry (details in the Supplementary Methods).

### Behavioral and histological analyses

The observer-blind measurements were performed before and after MCAO for a total of 5 weeks. The grip strength assessment was slightly modified from a previous report^65^ using an electronic device (MK-380CM/R, Muromachi kikai LTD). The rats were allowed to grasp a bar while being pulled with increasing force until they loosened their grip, and that grip strength was recorded. For the cylinder test, which was modified from a previous report^66^, the forelimb-use bias was analyzed during rearing movements and wall exploration in a transparent Plexiglas cylinder (diameter 18 cm, height 30 cm). Further details and histological analyses were described in the Supplementary Methods.

### OGD of cortical cells and transmembrane co-cultures with different stem cell types

Enriched neuronal cultures were obtained from E17 Sprague-Dawley rat cortices as described in a previous protocol^67^ and cultured in Neurobasal medium supplemented with 10% B27 (Gibco), 100 U/ml penicillin, and 100 mg/ml streptomycin with 5% CO_2_. For OGD, on day 5 the cultures were placed in a hypoxic incubator (Thermo Scientific model 3130) with 1% O_2_ with a gas mixture of 95% N_2_/5% CO_2_, and the medium was replaced by glucose-free DMEM (Gibco 11966-025) for the next 24 hours. Afterward, the cultures were returned to the normoxic incubator, and the medium was replaced with Neurobasal medium with B27. For the transmembrane co-cultures, the human iPSCs, iPSC-NPCs, BM-MSCs or WJ-MSCs (details in Supplementary Methods) were plated on membrane inserts at a density of 5 × 10^4^ cells per 10-mm insert (Nunc, with 0.2-µm pores) and placed above the rat cortical cultures immediately after OGD for 1–3 days prior to fixation (n = 6 per condition in each group). To investigate the beneficial effects of the candidate cytokines from the iPSC-NPCs, the OGD-subjected cortical cells were treated with individual cytokines or with a cocktail of the 5 cytokines. Alternatively, the co-cultures with iPSC-NPCs were treated with the 5 neutralizing antibodies (see Supplementary Table 1) for 72 hours at concentrations of 10 ng/ml and 100 ng/ml (n = 4 per concentration per cytokine).

### RNA extraction, cDNA preparation, and quantitative real-time PCR analysis

Using the RNeasy Mini Kit (Qiagen), total cellular RNA was extracted from the co-cultured human iPSCs, iPSC-NPCs, and BM-MSCs, as well as the rat OGD-subjected cells alone and the rat OGD-subjected cells co-cultured with iPSCs, iPSC-NPCs or BM-MSCs (details in Supplementary Methods, and Table 2 for primer sequences).

### Array data sets, array probe preparation and data processing

Published iPSC and MSC array data were downloaded from the Gene Expression Omnibus database (accession numbers GSE9709, GSE9832, GSE12390 and GSE13828). cRNA probes were prepared for an array hybridization, and the data analysis was performed as previously described^36, 37^. The Affymetrix™ human genome U133 Plus 2.0 and The Affymetrix™ rat genome 230 2.0 whole genome chips were used (GEO accession GSE82101) (details in Supplementary Methods).

### Statistical analysis

SPSS (Windows version 16.0.0) was used for all data analyses. A repeated-measures analysis of variance (ANOVA) test was used for the within-subject factor “TIME” and between-subjects factor “GROUP” to determine any significant differences in the behavioral measures, followed by Student’s *t*-tests with the Bonferroni correction at the different time points. To evaluate differences in the histological analyses between groups at 4 weeks post-MCAO, a Student’s *t*-test was performed. The required *in vivo* sample sizes (n = 9 per group) were calculated, assuming a treatment effect of 30-gf increase of grip strength with a 5% significance level and 90% power. For the OGD experiments, a one-way ANOVA test was used to evaluate the differences in the cell number, total nerve length, cytokine array densities, and gene expression levels among the multiple groups, followed by Tukey’s post hoc test. A *P*-value < 0.05 was considered to be statistically significant.

## Electronic supplementary material

Supplementary_methods, tables and figures
