# Supplementary material for: Delayed epidural transplantation of human induced pluripotent stem cell-derived neural progenitors enhances functional recovery after stroke
Source: Sci Rep. 2017 May 16;7:1943. doi: 10.1038/s41598-017-02137-w (PMC5434043; doi:10.1038/s41598-017-02137-w)

Delayed epidural transplantation of human induced pluripotent stem cell-derived neural progenitors enhances functional recovery after stroke

I-Hui Lee^1, 2^, Shiang-Suo Huang^3, 4^, Ching-Yu Chuang^5^, Ko-Hsun Liao^1^, Li-Hsin Chang^2^, Chia-Chi Chuang^1^, Yu-Shih Su^2^, Hung-Jui Lin^6^, Jui-Yu Hsieh^6^, Shu-Han Su^6^, Oscar Kuang-Sheng Lee^7, 8, 9^**, Hung-Chih** Kuo^5^,*

*^1^Department of Neurology, Neurological Institute, Taipei Veterans General Hospital, Taiwan**; ^2^Institute of Brain Science, National Yang-Ming University, Taipei, Taiwan; ^3^Department of Pharmacology and Institute of Medicine, Chung-Shan Medical University, Taichung, Taiwan;* *^4^Department of Pharmacy, Chung Shan Medical University Hospital, Taichung, Taiwan; ^5^Stem Cell Program, Institute of Cellular and Organismic Biology and Genomics Research Center, Academia Sinica, Taipei, Taiwan; ^6^Institute of Microbiology and Immunology, National Yang-Ming University, Taipei, Taiwan; ^7^Institute of Clinical Medicine, National Yang-Ming University, Taipei, Taiwan; ^8^Department of Orthopaedic Surgery, Taipei City Hospital, Taipei, Taiwan; ^9^Stem Cell Research Center, National Yang-Ming University, Taipei, Taiwan*

***Corresponding author:**

Hung-Chih Kuo, Ph.D.

No. 128, Sec. 2, Academia Road, Nankang, Taipei 11529, Taiwan

Tel: 886-2-27899580 ext.201

E-mail: [kuohuch@gate.sinica.edu.tw](mailto:kuohuch@gate.sinica.edu.tw)

**SUPPLEMENTARY MATERIALS AND METHODS**

***Derivation and culture of iPSCs***

Foreskin fibroblasts were plated in Dulbecco’s modified Eagle’s medium (DMEM) with 10% fetal bovine serum (FBS). The next day, the lentivirus expressing the retroviral receptor Slc7a1 (Addgene, Cambridge, MA) and Virapower packaging mix (Invitrogen) were added to the culture medium, and Slc7a1-expressing cells were selected with blasticidin. Plat-E cells (Cell Biolabs, Inc. Japan) with 1 µg/ml puromycin and 10 μg/ml blasticidin were transfected with pMXs-hOct4, pMXs-hSOX2, pMXs-hKlf4, or pMXs-hc-Myc (Addgene) using Fugene 6 (Roche). The Slc7a1-expressing human fibroblasts were then transfected with supernatant that contained the four retroviruses and polybrene. The transfected fibroblasts were re-plated onto mitomycin-C-inactivated MEFs in the fibroblast medium for an additional 5 d, and the medium was replaced with embryonic-stem-cell (ESC) medium (DMEM/F12 medium supplemented with 20% knockout serum replacement, Invitrogen), 1 mM L*-*glutamine, 1% non*-*essential amino acids, 0.1 mM β-mercaptoethanol*,* and 4 ng/ml recombinant human basic fibroblast growth factor (bFGF, Sigma). The cell colonies with ESC-like morphology were selected manually and were propagated in ESC culture conditions. The medium was changed daily and colonies were split every 6-7 d. For *in vivo* formation of iPSC teratomas, 1 × 10^6^-1 × 10^7^ iPSCs were intramuscularly injected into the rear legs of 5-8 week-old NOD-SCID mice. Teratomas were allowed to develop for 10-12 weeks after injection; they were subsequently excised and fixed overnight at 4°C in 4% paraformaldehyde, followed by cryosectioning and hematoxylin and eosin (H&E) staining. For flow cytometric analysis, cells were prepared according to the manufacturer’s instructions (BD FACSCalibur™ platform).

###### *Behavioral and histological analyses*

For the cylinder test, the first forelimb to be placed on the wall was scored for that limb. If this was immediately followed by the placement of the other limb on the wall without the removal of the first limb, then the movement was scored for both of the limbs. Each subsequent wall exploration was scored in the same manner for 3-5 min per rat. The average forelimb-use bias was calculated as: (movements using the right non-affected forelimb – the movements using the left affected forelimb) / the total number of forelimb movements.

The animals were euthanized intraperitoneally by an overdosed of chloral hydrate at four weeks post-MCAO. To visualize the viable versus infarct tissues, fresh brains were removed (n = 3 for the iPSC-NPCs group; n = 4 for the control group), sectioned into standard coronal slices using a brain matrix slicer (Jacobowitz Systems, Zivic-Miller Laboratories INC, Allison Park, USA), immersed in a 2% solution of the vital dye 2,3,5-triphenyltetrazolium chloride (TTC, Sigma) at 37°C in the dark for 30 min, and fixed with 10% formalin at room temperature overnight. For the histological analysis (n = 6 per group), the animals were intracardially perfused with saline containing 10 U/ml heparin followed by 4% paraformaldehyde in 0.1 M PBS. The brain was coronally cryosectioned at 20 µm thickness. The infarct/atrophy area was estimated by subtracting the area of the affected hemisphere from the area of the unaffected hemisphere, as shown by H&E staining using Image Pro Plus software V6.3 (Media Cybernetics, Inc., USA). The infarct/atrophy volume was estimated as the sum of 6 systematically sampled sections per animal, with an interval between sections of 1 mm and sampling tissue between 3 and -3 mm relative to bregma, which covered the infarct area^1^. The immunohistochemical quantification (see Table 1 for primary antibodies) was averaged from the aforementioned 6 sections per rat using Image Pro Plus software V6.3. The immunoreactive density was measured as the positive reactive area above a predetermined fluorescence intensity threshold divided by the total area^2^.

###### *Immunochemical studies*

Fixed cells on poly-D-lysine hydrobromide (PDL, P6407, Sigma)-coated coverslips, or cryosections were incubated in a blocking solution containing 0.15% Triton X-100 and 5% bovine serum albumin (BSA) in 0.1 M PBS at room temperature for 1 h. The samples were then incubated with primary antibodies (see Supplementary Table 1) at 4 ˚C overnight, washed and incubated with secondary antibodies conjugated with Alexa 488 and/or Cy3 (Jackson ImmunoResearch, 1:200) at room temperature for 1 h in the dark, mounted with VECTASHIELD mounting medium containing DAPI (H-1200, Vector Laboratories), and photographed using a confocal laser-scanning microscope (Olympus FLUOVIEW FV1000). For estimation of cell necrosis and apoptotic DNA fragmentation, propidium iodide staining (1:500), Terminal transferase dUTP Nick End Labeling (TUNEL) assays and Hoechst staining (1:1000) were performed.

***MSC cultures from bone marrow and umbilical cord Wharton’s Jelly***

Human bone marrow-derived MSCs (BM-MSCs) were cultured as previously reported. ^3^ Bone marrow aspirates from the iliac crest of healthy donors were washed with phosphate-buffered saline (PBS), loaded onto a Percoll solution, and centrifuged. The cells were then expanded in MesenPRO RS ^TM^ medium and growth supplement (Gibco 12746-012) with 100 U/ml penicillin and 100 mg/ml streptomycin. For mesenchymal tissues isolated from umbilical cords, tissues were diced into cubes in PBS and digested with collagenase and trypsin. The dissociated cells were plated on culture dishes in DMEM containing 10% FBS for expansion. For co-cultures, the cells were washed with PBS, trypsinized with 0.25% trypsin for 3 min at 37 ˚C, centrifuged, resuspended, and adjusted to aliquots of a concentration of 5 × 10^4^ cells/µl for use.

###### *RNA extraction, cDNA preparation, and quantitative real-time PCR analysis*

###### The concentration and purity of RNA were determined by measuring optical absorbance at 260 and 280 nm. Reverse transcription (RT) of cDNA was performed using the First cDNA Synthesis Kit (Fermentas) with 0.1-1 g of total RNA as template. The RT mixtures were used as templates in subsequent polymerase chain reaction (PCR). For quantitative real-time PCR (qPCR) analysis, the pre-messenger RNA sequence was obtained from the National Center for Biotechnology Information (NCBI) AceView program. All primers were designed with the Vector NTI Advance sequence analysis software (Invitrogen). RT-qPCR amplification of DNA fragments was conducted in MaximaTM SYBR Green qPCR Master Mix (Fermentas). The PCR products were detected and analyzed using a StepOneTM sequence detector (Applied Biosystems). The expression level of each gene was normalized to the expression level of glyceraldehyde 3-phosphate dehydrogenase (GAPDH).

***Array data sets, array probe preparation and data processing***

RNA log expression units were calculated from Affymetrix GeneChip array data using the ‘affy’ package of the Bioconductor suite of software for the R statistical programming language. Significant differences between sample groups were identified using the Bioconductor ‘*limma*’ package according to the previously described methods ^4^. To control for the multiple testing errors, a false discovery rate (FDR) algorithm was then applied to these *P*-values to calculate a set of *q*-values, i.e., thresholds of the expected proportion of false positives or false rejections of the null hypothesis. Heat maps were created using the dChip software. Principle component analysis (PCA) was performed using the Partek Genomics Suite to demonstrate how the various sample groups were related. Gene annotation was performed with the ArrayFusion web tool ^5^. A Gene Ontology database search was performed using the DAVID Bioinformatics Resources 6.7 interface ^6^. For functional regulatory networks, filtrated features from array analyses were subjected to the Ingenuity Pathway analysis (IPA) software to query all defined interactions of a given protein in the literature^7^.

***Cytokine antibody arrays and immunoblot analyses***

Antibody array experiments were repeated in triplicate. Relative hybridization signals were quantified using the ImageQuant software, and the duplicate spots per cytokine per array membrane were averaged. Those proteins with more than a 2-fold increase in co-cultures compared to the OGD-subjected cultures alone were considered significant and were further evaluated by RT-qPCR from lysates of human stem cells and rat OGD-injured cells to determine cell origin. The cells were lysed using radioimmunoprecipitation assay (RIPA) buffer containing 50 mM Tris, pH 7.5, 150 mM NaCl, 1% Nonidet-P40, 1% sodium deoxycholate, 0.1% sodium dodecyl sulfate (SDS) with protease inhibitors. The cell lysates were heated and resolved by electrophoresis in SDS-polyacrylamide gels for 2 h and electrotransferred onto Hybond-C extra membranes for 0.5 h (Amersham Biosciences). These membranes were blocked with 5% non-fat dry milk powder and 0.05% tween-20 in Tris buffered saline (TBS) at room temperature for an hour, followed by incubation with primary antibodies (Supplementary Table 1) in TBS at 4 °C overnight, then washed and incubated with horseradish peroxidase-conjugated secondary antibodies (Jackson ImmunoResearch) at room temperature for 2 h. GADPH or β-actin was used as the positive control for every membrane. The proteins of interest were visualized using the Western Lightning chemiluminescence reagent (PerkinElmer Life Sciences) and ECL Plus Western Blotting Detection Reagents (GE Healthcare). The film was exposed for a time between 30 s and 60 min, and the image displayed with ImageQuant LAS 4000 biomolecular imager (GE Healthcare).

**Supplementary References**

1 Paxinos, G. & Watson, C. The rat brain in stereotaxic coordinates. *Academic Press, London, 3rd ed.* (1997).

2 Lin, C. S. *et al.* Chronic intrathecal infusion of minocycline prevents the development of spinal-nerve ligation-induced pain in rats. *Regional anesthesia and pain medicine* **32**, 209-216 (2007).

3 Lee, O. K. *et al.* Fluvastatin and lovastatin but not pravastatin induce neuroglial differentiation in human mesenchymal stem cells. *J Cell Biochem* **93**, 917-928 (2004).

4 Storey, J. D. & Tibshirani, R. Statistical methods for identifying differentially expressed genes in DNA microarrays. *Methods Mol Biol* **224**, 149-157 (2003).

5 Yang, T. P., Chang, T. Y., Lin, C. H., Hsu, M. T. & Wang, H. W. ArrayFusion: a web application for multi-dimensional analysis of CGH, SNP and microarray data. *Bioinformatics* **22**, 2697-2698 (2006).

6 Dennis, G., Jr. *et al.* DAVID: Database for Annotation, Visualization, and Integrated Discovery. *Genome Biol* **4**, P3 (2003).

7 Huang, T. S. *et al.* Functional network reconstruction reveals somatic stemness genetic maps and dedifferentiation-like transcriptome reprogramming induced by GATA2. *Stem Cells* **26**, 1186-1201 (2008).

**Supplementary Table 1.** Summary of the used antibodies and cytokines

|  | Company | Number | Ratio |
| --- | --- | --- | --- |
| ***Cytokine*** | | | |
| BMP7 | R&D System | 354-BP |  |
| CXCL14 | Abcam | Ab50043 |  |
| FGF8 | Abcam | Ab50128 |  |
| FGF9 | Abcam | Ab50034 |  |
| IGFBP2 | Abcam | Ab63223 |  |
| ***Neutralizing antibodies*** | | | |
| BMP7 | R&D System | MAB3541 |  |
| CXCL14 | Peprotec | 500P237T |  |
| FGF8 | R&D System | MAB323 |  |
| FGF9 | R&D System | MAB273 |  |
| IGFBP2 | R&D System | Af674 |  |
| ***Immunoblot antibodies*** | | | |
| c-Jun | Abcam | Ab31419 |  |
| EGFR | Abcam | Ab2430 |  |
| ERK | BD | 610030 |  |
| GAPDH | Millipore | MAB374 |  |
| IkB | SantaCruz | sc-371 |  |
| NFκB | Millipore | 06-418 |  |
| Notch1 | Abcam | Ab8925 |  |
| Phos-c-Jun(AP-1) | Abcam | Ab13671 |  |
| Phos-EGFR | Abcam | Ab24912 |  |
| Phos-ERK | Millipore | 05-797R |  |
| Phos-P38 | BD | 612280 |  |
| Phos-NFkB | GeneTex | GTX107678 |  |
| ***Immunochemical anti-human antibodies*** | | | |
| ß-Tubulin type III | Chemicon |  | 1:500 |
| glial fibrillary astrocytic protein (GFAP) | Dako |  | 1:150 |
| Gamma Aminobutyric Acid (GABA) | Chemicon |  | 1:500 |
| homeobox protein engrailed-1 (EN1) | DSHB |  | 1:50 |
| insulin gene enhancer protein (ISLET1) | DHSB |  | 1:50 |
| orthodenticle homeobox 2 (OTX2) | R&D System |  | 1:100 |
| octamer-binding transcription factor 4 **(**OCT4) | Santa Cruz |  | 1:200 |
| Paired box gene 6 (PAX6) | DSHB |  | 1:50 |
| stage-specific embryonic antigen-4 (SSEA4) | Chemicon |  | 1:200 |
| sex determining region Y-box 1 (SOX1) | Chemicon |  | 1:100 |
| TRA-1-60 | Chemicon |  | 1:200 |
| Tyrosine hydroxylase (TH) | Chemicon |  | 1:500 |
| human nuclei | Chemicon | Mab1281 | 1:150 |
| ***Immunochemical anti-rat antibodies*** | | | |
| microtubule-associated protein 2 (MAP2) | Chemicon | Mab3418/Ab5622 | 1:75 |
| neuron-glial antigen 2 (NG2) | Abcam | Ab101807 | 1:100 |
| doublecortin (DCX) | Abcam | Ab18723 | 1:500 |
| ED1 (CD68) | ABD seroTec | Mca341R | 1:400 |
| glial fibrillary astrocytic protein (GFAP) | Dako | Z0334 | 1:500 |
| myelin basic protein (MBP) | Abcam | Ab40390 | 1:400 |
| rat endothelial cell antigen (RECA) | Abcam | Ab9774 | 1:50 |
| BrdU | Abcam | Ab1893 | 1:100 |

**Supplementary Table 2.** Summary of primer sequences used for RT-qPCR

| Gene |  | Sequence | Tm |
| --- | --- | --- | --- |
| hBMP2 | Forward | GCTTCCACCATGAAGAATCTTTGGA | 59.4 |
|  | Reverse | CCTGAAGCTCTGCTGAGGTGATAAA | 57.9 |
| hBMP7 | Forward | GACGCCCAAGAACCAGGAAGC | 59.6 |
|  | Reverse | CAGCCCAGGTCTCGGAAGCTG | 60.7 |
| hbeta-actin | Forward | CACCTTCTACAATGAGCTGCG | 54.0 |
|  | Reverse | TGCTTGCTGATCCACATCTGC | 54.0 |
| hFGF8 | Forward | CGACCCCTTCGCAAAGCTCA | 59.8 |
|  | Reverse | GCTTCCCCTTCTTGTTCATGCA | 57.9 |
| hFGF9 | Forward | ACACTGGAAGGCGATACTATGTTGC | 57.5 |
|  | Reverse | TACTTTGTCGGGGTCCACTGGTCT | 59.7 |
| hFGF18 | Forward | GCGAGGATGGGGACAAGTATGC | 59.3 |
|  | Reverse | GCCTTTGCGGTTCATGCACA | 59.1 |
| hGAPDH | Forward | ggAgTCCACTggCgTCTTCA | 55.9 |
|  | Reverse | TggTTCACACCCATgACgAA | 53.6 |
| hIGFBP1 | Forward | TCGTAGAGAGTTTAGCCAAGGCACA | 57.7 |
|  | Reverse | CATGGATGTCTCACACTGTCTGCTG | 59.3 |
| hIGFBP2 | Forward | GCCTGTACAACCTCAAACAGTGCAA | 57.7 |
|  | Reverse | GTAGAAGAGATGACACTCGGGGTCC | 61.0 |
| hIGF1 | Forward | CTGCTCACCTTCACCAGCTCTGC | 60.6 |
|  | Reverse | CCATACCCTGTGGGCTTGTTGAA | 57.1 |
| hMCP1 | Forward | GTGTCCCAAAGAAGCTGTGATCTTC | 57.7 |
|  | Reverse | TGGGTTGTGGAGTGAGTGTTCAAG | 57.4 |
| hMYC | Forward | GCGTCCTGGGAAGGGAGATCCGGAGC | 67.0 |
|  | Forward | CAACAACCGAAAATGCACCAGCCCCAG | 63.0 |
|  | Reverse | TTGAGGGGCATCGTCGCGGGAGGCTG | 67.0 |
| hNanog | Forward | AGTCCCAAAGGCAAACAACCCACTTC | 65.0 |
|  | Reverse | TGCTGGAGGCTGAGGTATTTCTGTCTC | 65.0 |
| hNTS | Forward | TGCTACTCCTGGCTTTCAGCTCC | 58.7 |
|  | Reverse | CAGAGTCATCTTCCAAGAGGGAACA | 57.6 |
| hOCT4 | Forward | CCCCAGGGCCCCATTTTGGTACC | 62.0 |
|  | Reverse | CTTCCCTCCAACCAG TTGCCCCAA AC | 63.0 |
| hSOX2 | Forward | GGGAAATGGGAGGGGTGCAAAAGAGG | 63.0 |
|  | Reverse | TTGCGTGAGTGTGGATGGGATTGGTG | 61.0 |
| hTERT | Forward | AGCTATGCCCGGACCTCCAT | 55.0 |
|  | Reverse | GCCTGCAGCAGGAGGATCTT | 55.0 |
| hWNT3 | Forward | GCGTGTTAGTGTCCAGGGAGTTCG | 60.7 |
|  | Reverse | GTGCATGTGGTCCAGGATAGTCGT | 58.8 |
| rCCL4 | Forward | ATGAAGCTCTGCGTGTCTGCCT | 57.8 |
|  | Reverse | AAGAGAAGCAGCAGGAAGTGGGA | 58.2 |
| rCCL7 | Forward | AGCTGCTGCTTTCACCGTGC | 57.8 |
|  | Reverse | CAGGGACACCGACTACTGGTGATCT | 59.3 |
| rCXCL10 | Forward | GTGCTGCTGTCGTTCTCTGCCT | 58.3 |
|  | Reverse | TCAGCGTCTGTTCATGGAAGTCG | 58.9 |
| rCXCL12 | Forward | TGTCCTCTTGCTGCCCAGCTCT | 60.0 |
|  | Reverse | GGCTGACTGGCTTACCGTCACTG | 59.7 |
| rCXCL16 | Forward | AGCACAAACAATGTGGAATCGG | 60.3 |
|  | Reverse | CGTGCTTGTGTCTGGAGGTT | 60.5 |
| rCXCR4 | Forward | ATGAAGCTCTGCGTGTCTGCCT | 64.5 |
|  | Reverse | AAGAGAAGCAGCAGGAAGTGGGA | 63.8 |
| rEGFR | Forward | CCACTGTGTCAAGACCTGCCCT | 57.7 |
|  | Reverse | GCCCAGCACATCCATAGGTACAGT | 58.1 |
| rFGFR1 | Forward | GCCCGAACAAGCTCAGCCCT | 60.2 |
|  | Reverse | CCGCAGCCAGTTGATGCTCT | 57.6 |
| rGAPDH | Forward | GTCGTGGAGTCTACTGGCGTCTTC | 60.8 |
|  | Reverse | CGTGGTTCACACCCATCACAAAC | 57.1 |
| rIGFBP1 | Forward | CAAGAAATGGAAGGAGCCCTG | 58.9 |
|  | Reverse | TCTTGTTGCAGTTTGGCAGA | 58.2 |
| rIGFBP2 | Forward | CCCAACTGTGACAAGCATGGC | 56.3 |
|  | Reverse | TGGATTGGCTTCCCAGTATTGG | 54.8 |
| rIGF1R | Forward | CGCACCAACGCTTCAGTTCCT | 56.3 |
|  | Reverse | CGCTGCCACCTCACAATGTAGTAAC | 59.3 |
| rIL1B | Forward | AGGCAGTGTCACTCATTGTGGCTG | 60.0 |
|  | Reverse | AGAGGACGGGCTCTTCTTCAAAGA | 58.6 |
| rITGA1 | Forward | AACAGGGCAAGGTGTACGTGTACG | 59.0 |
|  | Reverse | CCGCAGGGCTCATTCTTGTTT | 57.9 |
| rITGA5 | Forward | ATGTCCTCTACAAGCTCGGC | 65.1 |
|  | Reverse | TTCTGAGATCAGGAGGGCTCA | 61.5 |
| rITGB5 | Forward | ATCTCTTCTTCACTGCTACGTGCCA | 58.2 |
|  | Reverse | CGAGCCTCCACAGACACTTCAAAG | 59.0 |
| rMCP1 | Forward | TCAGCACCTTTGAATGTGAACTTGA | 54.4 |
|  | Reverse | AAGGCATCACATTCCAAATCACACT | 54.4 |
| rNotch1 | Forward | CCGCTGTGAGATTGATGTCAACG | 59.1 |
|  | Reverse | GGCACACTCGTCCGTGTTGATCT | 59.9 |
| rRet | Forward | GGCGAGTTTGGAAAAGTAGTCAAGG | 58.4 |
|  | Reverse | GCAGGTCTCGTAGTTCACTCTGGGA | 59.7 |
| rSfrp1 | Forward | CCACAACGTGGGCTACAAGAAGA | 57.9 |
|  | Reverse | CTTGTTGAGCAGCGGCACCC | 60.2 |
| rSfrp2 | Forward | AGCTCCCAAGGTGTGTGAAGCCT | 60.0 |
|  | Reverse | CTCCAGGATGATTTTGGTGTCTCTG | 57.8 |
| rSTAT2 | Forward | TGGAGCACCAGGATGATGATAA | 59.0 |
|  | Reverse | CCTGGTAGTGGCGGATGATT | 59.5 |
| rSTAT3 | Forward | GAACCTGGGACCAAGTGGC | 60.6 |
|  | Reverse | CTAAGAGCTTCTCGGCCAGC | 60.5 |

**Supplementary Figure 1.** (A) Raybio® Human Cytokine Antibody Array 6 with the supernatants from cultures of OGD-injured rat cortical cells alone, OGD-injured cells co-cultured with BM-MSCs or with iPSC-NPCs at 72 h post-OGD. Antibodies for each cytokine were duplicated on each membrane. Note that IGFBP2 was uniquely unregulated in the iPSC-NPCs co-cultures, whereas MCP1 was up-regulated both in BM-MSC and iPSC-NPC co-cultures but not in cultures of OGD-injured cells alone. (B) Quantification of the relative array signals of IGFBP2. (C) MCP1 and IGFBP2 mRNA levels were validated by RT-qPCR of stem cell lysates. IGFBP2 was overexpressed only in iPSC-NPCs. MCP1 was overexpressed in MSCs and in OGD-injured rat cells co-cultured with iPSC-NPCs but not in iPSC-NPCs alone (RT-qPCR confirmed).


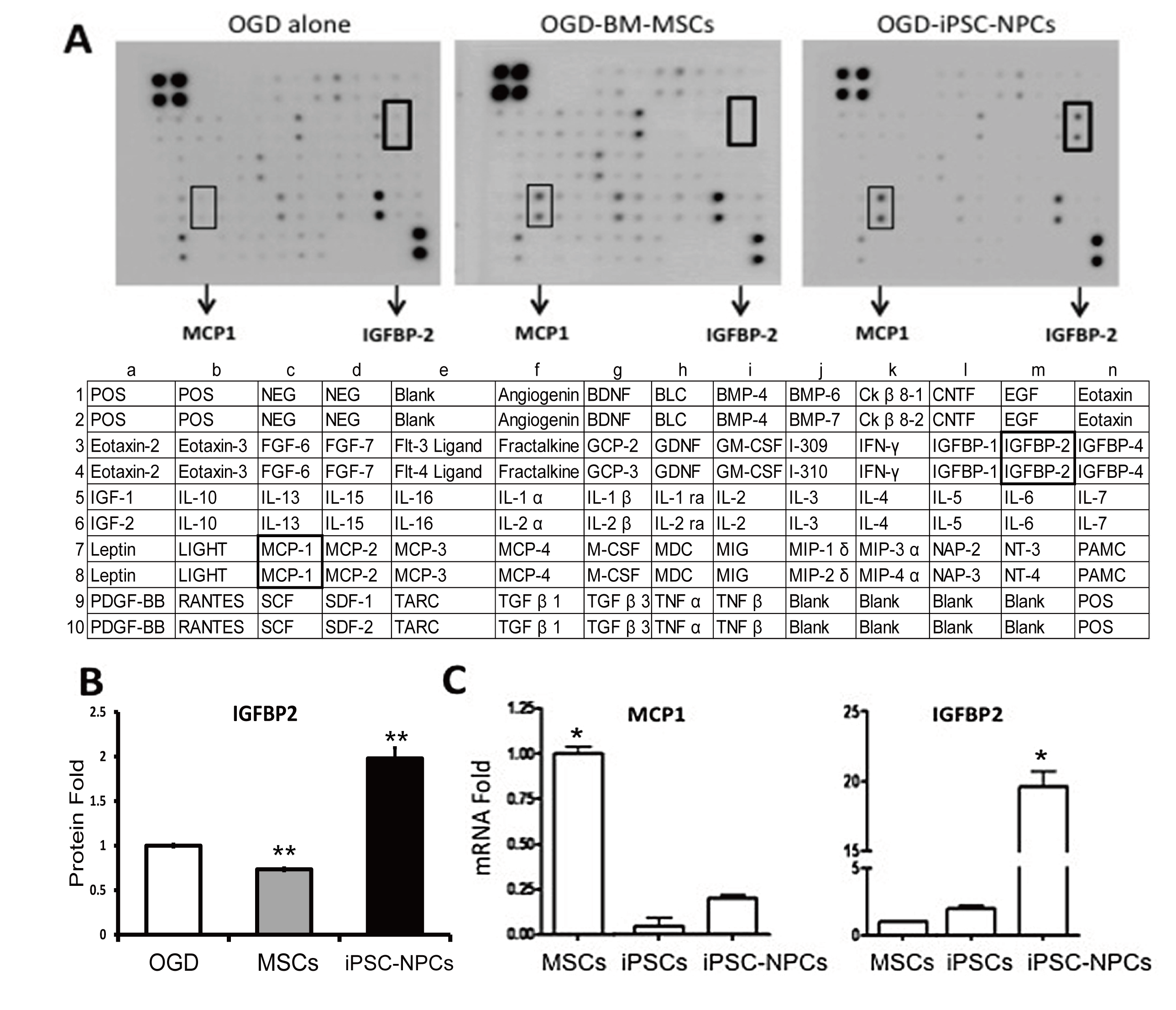


**Supplementary Figure 2.** Immunoblotting analyses were performed to confirm the protein expression of the candidate cytokines (BMP7, FGF8, FGF9, FGF12, CXCL14) identified from the enriched transcriptome of the iPSC-NPCs2. Neuroblastoma cells, SH-SY5Y, were used as a positive control.


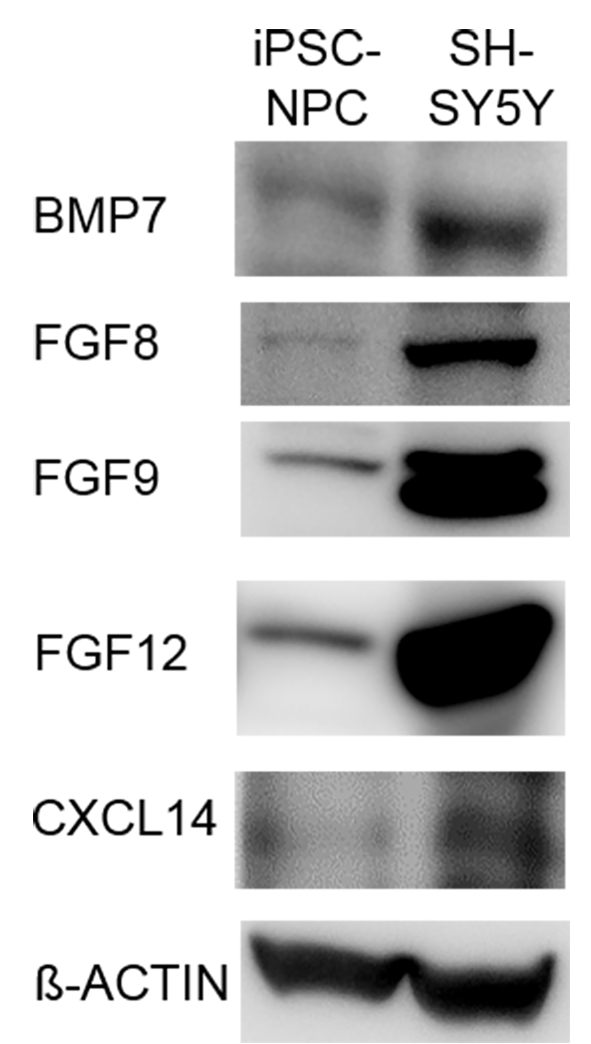

Supplement: Supplementary file 1 — Supplementary_methods, tables and figures [file 41598_2017_2137_MOESM1_ESM.doc]
